# Comparative effectiveness of Chuna manual therapy versus conventional usual care for non-acute low back pain: a pilot randomized controlled trial

**DOI:** 10.1186/s13063-019-3302-y

**Published:** 2019-04-15

**Authors:** Kyeong-Tae Lim, Eui-Hyoung Hwang, Jae-Heung Cho, Jae-Young Jung, Koh-Woon Kim, In-Hyuk Ha, Me-riong Kim, Kibong Nam, Min ho Lee A, Jun-Hwan Lee, Namkwen Kim, Byung-Cheul Shin

**Affiliations:** 1 Department of Rehabilitation Medicine of Korean Medicine, Pusan National University Korean Medicine Hospital, Yangsan, 50612 Republic of Korea; 2 Department of Korean Medicine, School of Korean Medicine, Pusan National University, Yangsan, 50612 Republic of Korea; 3 3rd Division of Clinical Medicine, School of Korean Medicine, Pusan National University, Yangsan, 50612 Republic of Korea; 4 Department of Rehabilitation Medicine of Korean Medicine, Kyung Hee University Korean Medicine Hospital at Gangdong, Seoul, 05278 Republic of Korea; 5 Jaseng Spine and Joint Research Institute, Jaseng Medical Foundation, Seoul, 06017 Republic of Korea; 6 Mokhuri Neck & Back Hospital, Repulic of, Seoul, 06272 South Korea; 7 Korean Medicine Life Science, University of Science & Technology (UST), Campus of Korea Institute of Oriental Medicine, Daejeon, 34054 Republic of Korea; 8 Center for Comparative Effectiveness Research & Economic Evaluation in Korean Medicine, Pusan National University, Yangsan, 50612 Republic of Korea

**Keywords:** Chuna manual therapy, Comparative effectiveness research, Low back pain, Pilot study, Randomized controlled trial

## Abstract

**Background:**

Low back pain (LBP) is common, with a lifetime prevalence of 80%, and as such it places substantial social and economic burden on individuals and society. Chuna manual therapy (CMT) combines aspects of physiology, biodynamics of spine and joint motion, and basic theory of movement dynamics. This study aimed to test the comparative effectiveness and safety of CMT for non-acute LBP.

**Methods:**

A three-arm, multicenter, pragmatic, randomized controlled pilot trial was conducted from 28 March 2016 to 19 September 2016, at four medical institutions. A total of 60 patients were randomly allocated to the CMT group (*n* = 20), usual care (UC) group (*n* = 20), or combined treatment (CMT + UC) group (*n* = 20), and received the relevant treatments for 6 weeks. The primary outcome was a numeric rating scale (NRS) representation of LBP intensity, while secondary outcomes included NRS of leg pain, Oswestry disability index (ODI), Patient Global Impression of Change (PGIC), the EuroQol-5 dimensions (EQ-5D), lumbar range of motion, and safety.

**Results:**

A total of 60 patients were included in the intention-to-treat analysis and 55 patients (CMT, 18; UC, 18; CMT + UC, 19) were included in the per-protocol analysis (drop-out rate 5.3%). Over the treatment period there were significant differences in the NRS score for LBP (CMT mean − 3.28 (95% CI − 4.08, − 2.47); UC − 1.95 (− 2.82, − 1.08); CMT + UC − 1.75 (− 2.70, − 0.80), *P* < 0.01) and the ODI scores in each group (CMT − 12.29 (− 16.86, − 7.72); UC − 10.34 (− 14.63, − 6.06); CMT + UC − 9.27 (− 14.28, − 4.26), *P* < 0.01). The changes in other secondary outcomes did not significantly differ among the three groups. Sixteen minor-to-moderate safety concerns were reported.

**Conclusions:**

Our results suggest that CMT has comparative efficacy for non-acute LBP and is generally safe. As this was a preliminary study, a well-powered (over 192 participants) two-arm (CMT versus UC) verification trial will be performed to assess the generalizability of these results.

**Trial registration:**

Clinical Research Information Service (CRIS), KCT0001850. Registered on 12 March 2016.

## Background

Low back pain (LBP) is common, with a lifetime prevalence of 80% and it places major social and economic costs on individuals and society among adults of working age [1, 2]. A total of 62% of people who experience LBP will develop chronic symptoms lasting longer than 1 year [3]. For this reason, there are various standard treatments for managing back pain [4]; however, patients are often dissatisfied with these treatments [5].

Spinal manual therapy (SMT) is one of the treatment options for acute, non-acute, and chronic LBP [6]. A number of studies, including systematic reviews and randomized controlled trials (RCTs), have shown that SMT is as effective as other therapies, such as exercising and standard medical care or physiotherapy [7], and it is recommended that SMT be used for acute and chronic LBP [8–10].

*Chuna,* which means manual treatment in Korean, is based on traditional Korean medicine theory, which includes meridian theory and anatomy with radiology-based diagnosis. Chuna manual therapy (CMT) is a sub-specialty that seamlessly brings together aspects of physiology, biodynamics of spine and joint motion, and the basic theory of movement dynamics. CMT is popular in South Korea, where other manipulation methods such as osteopathy, chiropractic and other manipulative therapies have been developed [11]. While the number of RCTs has been increasing, well-designed clinical studies that evaluate the effectiveness of CMT are rare, albeit that patients report treatment satisfaction overall [12, 13]. Furthermore, the use of CMT by patients is limited due to cost, because it is not covered by Korean National Health Insurance, unlike in other countries such as Germany [6, 14]. However, in 2011 the National Assembly Forum for Insurance Guarantee Reinforcement of Korean Medicine suggested that national insurance coverage of Korean medicine treatments, including CMT, should be increased, which would increase the accessibility of Korean medicine services [15]. On the basis of this medical plan, the Korean Ministry of Health and Welfare started a national insurance pilot project covering CMT in 65 traditional Korean medical institutions in 2017.

Against this background, it is therefore important to prove the comparative effectiveness and safety of CMT. On the basis of high-quality clinical evidence on CMT, we conducted a pilot study to explore the feasibility of using CMT for LBP, because of social demand, to determine the effectiveness and safety value of CMT in treating non-acute LBP to lay the basis for a future well-designed, high-quality RCT. To explore real-life clinical CMT treatment conditions, our first object was to conduct a pilot trial to test the comparative efficacy and safety of CMT as compared to conventional usual care (UC), by comparison of pain, functionality, and adverse events. Second, we compared the efficacy and safety of treatment involving a combination of CMT and UC (CMT + UC), as compared to CMT or UC alone, which reflects the situation in actual clinical practice.

## Methods

### Study design

This research adhered to Consolidated Standards of Reporting Trials (CONSORT) guidelines [16]. The Chuna Research Network (CRN) comprised four Korean medical institutions (two university-based Korean medicine hospitals and two spine specialty hospitals) and several expert discussions were conducted to devise a pilot protocol for conducting a trial (once a month). This multicenter, pragmatic, randomized controlled pilot trial, with three parallel arms, was designed to explore the feasibility of a trial to evaluate the clinical efficacy, safety, and cost-effectiveness of using CMT in patients with non-acute LBP. The study protocol was registered with the Clinical Research Information Service (CRIS identifier KCT0001850, 12 March 2016). Additionally, the protocol was approved by the institutional review board (IRB) of Pusan National University Korean Medicine Hospital on 11 March 2016 (IRB approval number 2016002) and had already been published [17]. After screening, participants were randomized into three groups (CMT, UC, and CMT + UC group) by central allocation and treated for 6 weeks consecutively. Other additional treatments (e.g., medications related to pain, acupuncture, procedures, or surgery) not specified in the protocol were not allowed during the 6-week intervention period. Study monitoring was carried out by the Contract Research Organization (CRO), which had no role in the research design and practice at each site.

### Subjects

The study was conducted in four major Korean medicine hospitals in Korea (Pusan National University Korean Medicine Hospital, Kyung Hee University Korean Medicine Hospital at Gangdong, Jaseng Hospital of Korean Medicine, and Mokhuri Neck and Back Hospital) from 28 March 2016, to 19 September 2016. Patients aged 19–70 years, with non-acute LBP, were considered on the basis of eligibility criteria. Patients were included in the study only when they met the following criteria: (1) non-acute LBP (with pain duration of 3 weeks or longer) requiring medical attention; (2) average numeric rating scale (NRS) score of more than 5 during the previous week; (3) aged from 19 to 70 years, inclusive; and (4) agreed to trial participation and provided written informed consent. Patients were excluded when they (1) were diagnosed with serious pathologic condition(s) that might cause LBP (e.g., spinal metastasis from tumor(s), acute fracture, spinal dislocation); (2) had undergone spinal surgery within the past 3 months; (3) were diagnosed with other chronic disease(s) that might interfere with the treatment effect or interpretation of the outcome (e.g., chronic renal failure); (4) were diagnosed with a progressive neurological deficit or had severe neurological symptoms; (5) had an inner fixation or stabilization device mounted through spinal surgery; (6) were currently taking steroids, immunosuppressants, medicine for psychological problems. or other medication(s) that might interfere with the study results; (7) had received CMT or medicine that may influence pain levels, such as nonsteroidal anti-inflammatory drugs (NSAIDs), within the past week; (8) were pregnant or were planning to become pregnant; or (9) were participating in other clinical studies or were otherwise deemed unsuitable by the researchers.

### Recruitment and randomization procedures

The exact procedures and details of this study have been published in a pilot protocol [17]. Briefly, the participants were recruited through advertisements, posters on hospital bulletin boards, and referrals from Korean Medicine doctors (KMDs) in hospitals. Potential participants were asked to answer questions and were evaluated by KMDs or by the clinical research coordinator to determine eligibility. If patients were eligible for trial participation in accordance with the inclusion and exclusion criteria, they were randomized per center and allocated to one of the three groups using block randomization (block size 3). A random sequence was generated by an independent statistician using SAS 9.3 (SAS Institute Inc., Cary, NC, USA). The participants enrolled at the four sites were randomly allocated to groups without stratification by site. Due to the dissimilarity of the interventions, blinding of physicians and participants to allocation of treatment groups was impossible, by nature of the interventions. Only outcome assessors, the statistician, and data analysts were blinded and conducted the outcome assessment in a separate room after treatments were performed by separate physicians. The electronic data that did not contain participants’ information or participants’ allocation were transferred to the statistician and data analysts. All allocations were concealed as far as possible.

### Interventions

#### Chuna manual therapy

Participants assigned to the CMT group received CMT administered by a qualified KMD with over 3 years of clinical experience of CMT and who received Chuna protocol training sessions using an established, semi-standardized Chuna treatment plan for LBP [18, 19]. CMT included various techniques, such as high-velocity, low-amplitude thrusts to spinal joints, slightly further than passive range of motion, and mobilization involving application of manual force to joints in the passive range of motions [20]. The detailed mandatory, selective, and regional CMT techniques in this study were described in the protocol [17]. A total of 10–18 CMT sessions were performed over two periods (minimum of 10 sessions); these periods involved 2–3 sessions/week in week 1 to week 4 and 1–3 sessions/week in week 5 to week 6. The duration of CMT treatment in a session was approximately 15 min, which included evaluation and administration.

#### Usual care

Participants assigned to the UC group were administered physiotherapy, oral medication, and 15-min structured education on LBP care. Conventional oral medication and physiotherapy were provided with reference to the most common treatments used in patients with LPB, as assessed from the 2011 Korean Health Insurance Review and Assessment (HIRA) statistics [21]. Participants were asked to record drug intake to monitor adherence, and medicine and physiotherapy usage type and frequency in a separate case report form. The duration and frequency of UC group treatment sessions were similar to those in the CMT group.

### Combined treatment with CMT and usual care

Participants assigned to the concurrent CMT and UC group received UC treatment in addition to CMT treatment. Treatments involved the same method, frequency, session length, total duration, and number of sessions as in the individual treatment groups.

### Outcomes

For the primary outcome, we measured NRS scores of LBP levels for the previous week. NRS scores ranged from 0 to 10, with the higher number indicating greater pain intensity. The secondary outcome included NRS scores for leg pain, evaluating functional status by using the Korean version of the Oswestry Disability Index (ODI) questionnaire [22]. The ODI questionnaire was used to measure LBP-related disability. It was composed of 10 questions, including questions on daily life, pain intensity, personal care, lifting, walking, sitting, standing, sleeping, social life, and travelling. The Patient Global Impression of Change (PGIC) was one of the secondary outcomes, which assessed comprehensive and global change in LBP and movement limitation due to pain [23]. The PGIC consisted of 7-level answers, where lower numbers indicated lower treatment satisfaction. The EuroQol-5 dimensions (EQ-5D) health survey was also used to assess secondary outcomes. The EQ-5D is composed of 5 dimensions assessing the current health state, consisting of mobility, self-care, usual activities, pain/discomfort, and anxiety/depression. Each dimension was evaluated by 3-level answers, with the lower score indicating the patient has a better state of health. Patients’ quality of life was assessed using the validated Korean version of the EQ-5D [24]. The Health Utility Index III (HUI-III), including sight, hearing, speaking, walking, agility, emotion, cognition, pain, and quality of life values was used to calculate participants’ quality of life as with the EQ-5D [25]. Lumbar range of motion (ROM) was also measured for assessing improvements objectively [26]. The maximum lumbar spine angle between a perpendicular line was measured on flexion, extension, lateral bending, and lateral rotation, using a goniometer. The angle was recorded as 0° if a patient complained that lumbar movement was impossible due to pain. A 9-point Likert-scale credibility and expectancy questionnaire was used to assess treatment expectation at the first visit. Cost data were also investigated in this study, but the results of these investigations will be reported in a separate paper. All participants were followed up at 1, 3, and 4 weeks after the 6-week treatment periods. At each visit the participant was assessed before treatment, to record the outcomes of the previous treatment session.

### Statistical analysis

Data were summarized using descriptive statistics: frequency was calculated as percentage for categorical variables and mean ± standard deviation (SD) was calculated for continuous variables. Differences in study participants’ characteristics were compared across subgroups using the chi-square test or Fisher’s exact test for categorical variables and analysis of variance or the Kruskal-Wallis test for continuous variables, as appropriate. The paired *t* test, independent *t* test, or Wilcoxon’s signed rank test were also employed to assess the differences between assessment points or between two groups. Analysis of covariance was employed to reduce error from inequality at baseline, using the baseline value as a covariate. We used the Shapiro-Wilk test to check whether the data distribution was normal. Intention-to-treat (ITT) and per-protocol (PP) analyses were performed and the last observed carried forward (LOCF) method was used to impute missing values. All statistical analyses were carried out using SPSS Statistics for Windows 22.0 (IBM Corp. Armonk, NY, USA) statistical software. All tests were two-tailed at the 5% significance level.

The sample size calculation method for this study was published in the protocol [17]. A significance level of 5% (α error), type 2 error of 20% (β error), power of 80%, and 25% drop-out rate were applied to the formula shown in the protocol; the number of participants required in total was 60 (20 per group). All statistical analyses were performed blinded and independently by a statistician.

### Safety

To monitor the safety of CMT, UC, and CMT + UC treatment, participants were asked about adverse events (AEs) at each visit. If AEs occurred, physicians rated the relationship between each treatment and the outcome on a 6-point scale (1 = definitely related; 2 = probably not related; 3 = possibly related; 4 = probably not related; 5 = definitely not related; and 6 = unknown) and categorized into 3 levels using the Spilker classification (mild, moderate, severe) [27, 28]. If serious adverse events (SAEs) occurred during the study, unblinding was considered allowable and the physician would inform the relevant IRB and main study site (Pusan National University Korean Medicine Hospital) to decide whether the trial should be continued or terminated. Participants suffering AEs would receive appropriate medical attention and damage compensation.

## Results

**Fig. 1:**
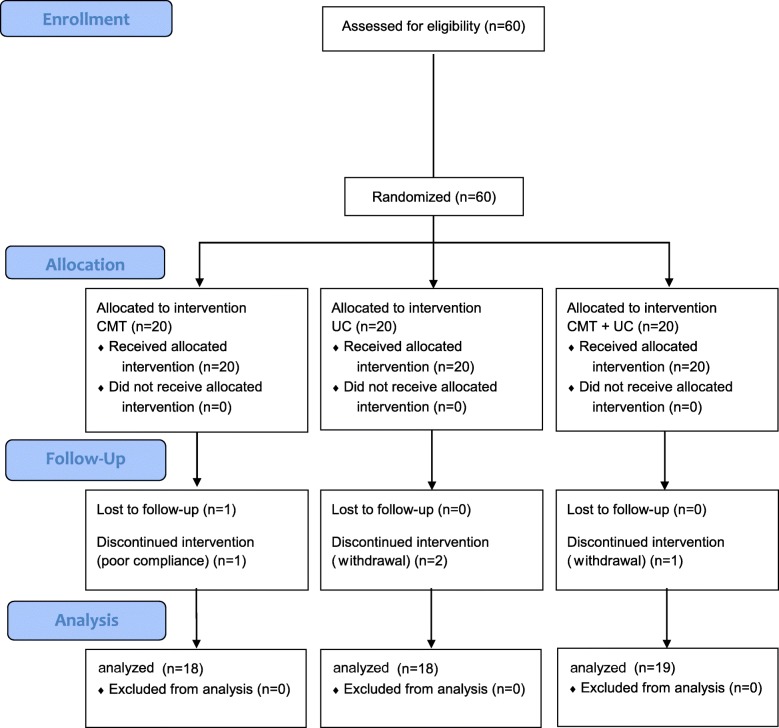
Consolidated Standards of Reporting Trials (CONSORT) 2010 flow diagram. CMT, Chuna manual therapy; UC, usual care

Figure 1 shows the flow of participants through the trial. There were 60 participants who responded to the recruitment materials, and 60 were eligible and allocated into three groups at four medical institutions, from 28 March 2016 to 19 September 2016. Four participants dropped out during the treatment period and another participant was eliminated after finishing all the treatments, because of loss to follow up. A total of 60 patients (20 per group) were analyzed as ITT, and 55 patients (18 in the CMT, 18 in the UC, and 19 in the CMT + UC group) were analyzed as PP. Five participants in four centers dropped out or were lost to follow up during the pilot study: four of these were due to withdrawal of consent, while the remaining participant was involved in another clinical trial in the follow-up term (drop-out rate 8.3%). We reported trial results by ITT analysis, following the previous protocol [17].

Table 1 shows the demographic features of the participants at baseline. There were no statistically significant differences among the three groups in terms of sex, age, height, or weight (*P* > 0.05), or in terms of expectation scale, symptom onset, mean NRS for LBP, ODI, EQ-5D, and ROMs of the lumbar spine at the evaluation in the first week, except for the NRS for leg pain (*p* = 0.018).

**Table 1:** Demographic features of the participants at baseline

Variables	Group			*P* value*
	CMT (*n* = 20)	UC (*n* = 20)	CMT + UC (*n* = 20)	
Sex, *n* (%)				1.000^a^
Male	5 (25.0)	5 (25.0)	4 (20.0)	
Female	15 (75.0)	15 (75.0)	16 (80.0)	
Age (years)	41.70 ± 13.27	37.80 ± 12.21	41.20 ± 12.86	.576^b^
Height (cm)	164.75 ± 7.22	165.45 ± 7.77	163.80 ± 7.72	.904^c^
Weight (kg)	63.60 ± 10.65	59.25 ± 12.05	61.10 ± 9.74	.450^b^
Symptom onset (years)	5.09 ± 4.32	4.97 ± 4.82	7.46 ± 7.57	.467^c^
NRS (LBP)	5.80 ± 1.32	5.50 ± 1.05	6.05 ± 1.15	.176^c^
NRS (leg pain)	3.60 ± 2.93	1.85 ± 2.46	4.40 ± 3.03	.018^c^
ODI	25.17 ± 6.52	26.06 ± 7.78	28.47 ± 8.87	.388^b^
EQ-5D (points)	0.70 ± 0.19	0.74 ± 0.06	0.63 ± 0.21	.078^c^
HUI-III	0.88 ± 0.12	0.88 ± 0.13	0.91 ± 0.09	.750^c^
ROM (flexion)	89.00 ± 9.40	89.30 ± 7.66	81.65 ± 18.26	.261^c^

All values are shown as mean ± standard deviation, except for sex. *Statistical analysis was conducted based on intension-to-treat analysis, imputing by the method of last observation carried forward. Shapiro-Wilks’s test was employed to test the normality of the data distribution

*CMT* Chuna manual therapy, *UC* usual care, *CI* confidence interval, *NRS* numerical rating scale, *LBP* low back pain, *ODI* Oswestry Disability Index, *PGIC* Patient’s Global Impression of Change, *EQ-5D* EuroQol 5 dimensions questionnaire, *HUI-III* Health Utility Index, *ROM* range of motion

^a^*P* values were derived from Fisher’s exact test

^b^*P* values were derived from analysis of variance with Scheffe’s method for pairwise comparison

^c^*P* values were derived from the Kruskal-Wallis test with the Mann-Whitney U test for pairwise comparison

The NRS scores for LBP, ODI, and EQ-5D statistically significantly improved (Table 2) at the primary endpoint (week 7) compared with the baseline data at week 1, in all three groups. Changes in NRS scores for LBP differed significantly between the CMT only and CMT + UC group, but other factors did not. In terms of changes in LBP NRS scores, ODI differed in the following order: CMT alone group, UC alone group, and CMT + UC group. Other outcomes, such as the NRS scores for leg pain in the CMT and the CMT + UC groups, flexion and left rotation ROM of the lumbar spine in the CMT + UC group, left lateral flexion in the UC group, and HUI-III in the CMT group differed significantly as compared with week 1. However, there were no statistically significant differences in the NRS of leg pain, ODI, EQ-5D, or HUT-III among the three groups at week 7 (Table 2).

**Table 2:** Comparison by treatment group at each assessment period

Variables	Group			*P* value*
	CMT (*n* = 20) Mean [95% CI]	UC (*n* = 20) Mean [95% CI]	CMT + UC (*n* = 20) Mean [95% CI]	
NRS (LBP)				
1st Week	5.80 [5.18, 6.42]	5.50 [5.01, 5.99]	6.05 [5.51, 6.59]	0.176^c^
7th Week	2.53 [1.78, 3.28]	3.55 [2.73, 4.37]	4.30 [3.25, 5.35]	0.016^b^
Difference	− 3.28 [− 4.08, − 2.47]	−1.95 [− 2.82, − 1.08]	−1.75 [− 2.70, − 0.80]	0.021^c^
*P* value	< 0.001^e^	< 0.001^f^	0.005^f^	
NRS (leg pain)				
1st Week	3.60 [2.23, 4.97]	1.85 [0.70, 3.00]	4.40 [2.98, 5.82]	0.018^c^
7th Week	1.15 [0.40, 1.90]	1.15 [0.45, 1.85]	2.55 [1.53, 3.57]	0.053^c^
Difference	− 2.45 [− 3.53, − 1.37]	−0.70 [− 1.73, 0.33]	−1.85 [− 3.10, − 0.60]	0.096^c^
*P* value	< 0.001^f^	0.140^f^	0.015^f^	
ODI				
1st Week	25.17 [22.12, 28.22]	26.06 [22.42, 29.70]	28.47 [24.55, 28.57]	0.388^b^
7th Week	12.88 [9.00, 16.75]	15.71 [11.39, 20.04]	19.20 [13.75, 24.65]	0.135^b^
Difference	− 12.29 [− 16.86, − 7.72]	−10.34 [− 14.63, − 6.06]	−9.27 [− 14.28, − 4.26]	0.933^c^
*P* value	< 0.001^e^	< 0.001^f^	0.001^e^	
PGIC				
1st Week	–	–	–	–
7th Week	6.00 [5.60, 6.39]	5.06 [4.62, 5.49]	5.53 [5.06, 5.99]	0.011^c^
EQ-5D (points)				
1st Week	0.70 [0.62, 0.79]	0.74 [0.71, 0.76]	0.63 [0.53, 0.73]	0.078^c^
7th Week	0.88 [0.79, 0.92]	0.81 [0.75, 0.87]	0.77 [0.70, 0.84]	0.180^c^
Difference	0.16 [0.06, 0.25]	0.07 [0.01, 0.14]	0.14 [0.03, 0.25]	0.421^b^
*P* value	0.002^f^	0.033^f^	0.019^f^	
HUI-III				
1st Week	0.88 [0.82, 0.93]	0.88 [0.82, 0.94]	0.91 [0.87, 0.95]	0.750^c^
7th Week	0.95 [0.92, 0.98]	0.93 [0.89, 0.97]	0.91 [0.86, 0.95]	0.233^c^
Difference	0.07 [0.02, 0.17]	0.05 [−0.01, 0.11]	0.00 [− 0.04, 0.03]	0.037^c^
*P* value	0.011^f^	0.054^f^	0.857^f^	
ROM (flexion)				
1st Week	89.00 [84.60, 93.40]	89.30 [85.71, 92.89]	81.65 [73.10, 90.20]	0.261^c^
7th Week	94.00 [90.01, 97.99]	88.50 [83.35, 93.66]	88.00 [79.60, 96.40]	0.253^c^
Difference	5.00 [−0.68, 10.68]	−0.80 [− 3.77, 2.17]	6.35 [− 0.46, 13.16]	0.026^c^
*P* value	0.063^f^	0.579^e^	0.049^f^	

All values are mean ± standard deviation. *Statistical analysis was conducted based on intension-to-treat analysis, imputing by the method of last observation carried forward.

*CMT* Chuna manual therapy, *UC* usual care, *CI* confidence interval, *NRS* numerical rating scale, *LBP* low back pain, *ODI* Oswestry Disability Index, *PGIC* Patient’s Global Impression of Change, *EQ-5D* EuroQol 5 dimensions questionnaire, *HUI-III* Health Utility Index, *ROM* range of motion

^a^*P* values were adjusted for observed value at baseline by using baseline value as covariate in analysis of covariance

^b^*P* values were derived from analysis of variance

^c^*P* values were derived from the Kruskal-Wallis test

^d^*P* values were derived from the paired *t* test

^e^*P* values were derived from the Wilcoxon signed-rank test

There were 16 minor-to-moderate AEs that occurred during the trial, but there were no significant differences among the three groups in the frequency of AEs.

## Discussion

Many people suffer from LBP and spend large amounts of money to relieve pain by using conservative medical treatment [6]. Various conservative treatments, including manipulative therapy, which are recommended for and are used to treat non-acute LBP in several clinical practice guidelines and systematic reviews, but the evidence for individual usual care was insufficient [8–10]. CMT is a Korean-style manipulative therapy that is widely used for managing acute, sub-acute, or chronic LBP in South Korean patients, who have reported satisfaction with the treatment [13]. However, there has been a shortage of well-designed clinical studies supporting its use. Therefore, we conducted a pilot study to explore the feasibility of a study to evaluate the effectiveness and safety of CMT and to guide the design of a future better-powered full-scale RCT as a national funding project.

Here we focused on calculating sample size and analyzing the validity of a future trial that could determine the actual effectiveness of CMT. We further observed whether CMT alone is more effective than UC alone for non-acute LBP in this regard. We found no statistically significant differences among the three groups, but on post-hoc comparison the changes in NRS scores for LBP in the CMT alone group (mean − 3.28, 95% confidence interval − 5.01, − 1.55) differed from those in the UC alone group (mean − 1.75, 95% confidence interval − 3.77, 0.27) at week 7 as compared to week 1.

Furthermore, the ODI score differed among the three groups. Changes in the mean ODI favored the use of CMT alone in terms of functional improvement in patients with LBP; this is plausible, as improvement in lumbar spine function is strongly related to pain relief.

However, the CMT + UC group showed the least improvement in terms of some outcomes, including changes in NRS scores for LBP and ODI. Therefore, we assumed that differences among the three groups represent a limitation of our pilot study, reflecting participants’ expectations and the bias caused by dissimilar interventions. Although the participants were recruited as having “chronic low back pain” according to the inclusion/exclusion criteria of this study, “chronic back pain” may also be referred to as “recurrent low back pain” when the pain episode period has continued for 3 months or longer. So, the fact that it is difficult to discern whether the pain reduction was due to the therapeutic effect or remission of “recurrent low back pain” is a major limitation of this study. However, it can be assumed that the limitations of measuring the effectiveness of treatment in the midst of the pain fluctuations in “recurrent low back pain” is applicable to all three groups (CMT alone, UC alone, CMT + UC) in this pragmatic RCT design, which compares UC and CMT + UC, and we also followed the patients for 8 weeks following the 6-week treatment period, which would allow us to check for pain fluctuations in this short-term period. We plan to perform a full-scale RCT using CMT + UC versus UC alone in a full-scale, two-arm study. We also calculated the appropriately powered sample size for a two-arm model. Based on the *t* test, with a 5% significance level, 80% power, and a 20% drop-out rate, the SD of the NRS score between two groups was presumed to be 3.3, based on an ITT analysis. According to these sample size calculations, a total of 194 participants (97 per group) would need to be recruited for this future trial. With such a two-arm model and the calculated sample size, a future verification trial would be better suited to evaluate the effectiveness and safety of using CMT in combination with UC for managing non-acute LBP in the real-life Korean clinical condition.

## References

[1] Juch JNS, Mas ET, Ostelo RWJG (2017). Effect of radiofrequency denervation on pain intensity among patients with chronic low back pain: the mint randomized clinical trials. JAMA.

[2] Hoy D, March L, Brooks P (2014). The global burden of low back pain: estimates from the Global Burden of Disease 2010 study. Ann Rheum Dis.

[3] Hestbaek L, Leboeuf-Yde C, Manniche C (2003). Low back pain: what is the long-term course? A review of studies of general patient populations. Eur Spine J.

[4] Deyo RA, Weinstein JN (2001). Low back pain. N Engl J Med.

[5] Lee JH, Park HJ, Lee H (2010). Acupuncture for chronic low back pain: protocol for a multicenter, randomized, sham-controlled trial. BMC Musculoskelet Disord.

[6] Walker J, Mertens UK, Schmidt CO (2017). Effect on healthcare utilization and costs of spinal manual therapy for acute low back pain in routine care: a propensity score matched cohort study. PLoS One.

[7] Rubinstein SM, van Middelkoop M, Assendelft WJJ, et al. Spinal manipulative therapy for chronic low-back pain. Cochrane Database Syst Rev. 2011, Issue 2. Art. No.: CD008112. 10.1002/14651858.CD008112.pub2 PMC12009663 21328304

[8] Koes BW, van Tulder M, Lin CW (2010). An updated overview of clinical guidelines for the management of non-specific low back pain in primary care. Eur Spine J.

[9] Chou R, Qaseem A, Snow V (2007). Diagnosis and treatment of low back pain: a joint clinical practice guideline from the American College of Physicians and the American Pain Society. Ann Intern Med.

[10] van Tulder M, Becker A, Bekkering T (2006). Chapter 3. European guidelines for the management of acute nonspecific low back pain in primary care. Eur Spine J.

[11] Park JM, Shin SW, Park JH (2008). A comparative study on the concepts of the Chuna (推拿). J Korean Med Classics.

[12] Hwang MS, Cho HW, Lee HY, et al. Research trends on Chuna treatment in Korean Medicine: Focused on type of clinical trials, published year, academic journals and treatment technique for each used parts. J Korea Chuna Manual Med Spine Nerves. 2013;8(1)49–61.

[13] Cho JG, Kim NS, Do SR, et al. National survey on the use of Korean medicine and Korean herbal medicine. Seoul: Korea Ministry of Health and Welfare, Korea Institute for Health and Social Affairs; 2011. 11-1352000-000547-12.1-554.

[14] Park J, Kwon SM (2011). Determinants of the utilization of oriental medical services by the elderly. J Korean Oriental Med.

[15] Ko Y, Lee J, Hwang E, et al. A study to prepare the health insurance coverage for Chuna manual therapy. J Korea Chuna Manual Med Spine Nerves. 2012;7(2):1–14.

[16] Boutron I, Altman DG, Moher D (2017). CONSORT statement for randomized trials of nonpharmacologic treatments: a 2017 update and a CONSORT extension for nonpharmacologic trial abstracts. Ann Intern Med.

[17] Shin BC, Kim MR, Cho JH (2017). Comparative effectiveness and cost-effectiveness of Chuna manual therapy versus conventional usual care for nonacute low back pain: study protocol for a pilot multicenter, pragmatic randomized controlled trial (pCRN study). Trials.

[18] Kim B, Hwang E, Heo K (2015). The survey on the standardization of Chuna manual technique for operating RCT of non-acute low back pain. J Korea Chuna Manual Med Spine Nerves.

[19] Shin YS, Shin JS, Lee J (2015). A survey among Korea Medicine doctors (KMDs) in Korea on patterns of integrative Korean Medicine practice for lumbar intervertebral disc displacement: preliminary research for clinical practice guidelines. BMC Complement Altern Med.

[20] Park JJ, Shin J, Choi Y (2010). Integrative package for low back pain with leg pain in Korea: a prospective cohort study. Complement Ther Med.

[21] Health Insurance Review and Assessment Service. 2017 [cited 2017 5 Jan]; Available from: http://www.hira.or.kr/.

[22] Jeon CH, Kim DJ, Kim SK (2006). Validation in the cross-cultural adaptation of the Korean version of the Oswestry Disability Index. J Korean Med Sci.

[23] Farrar JT, Young JP, LaMoreaux L (2001). Clinical importance of changes in chronic pain intensity measured on an 11-point numerical pain rating scale. Pain.

[24] Kim MH, Cho YS, Uhm WS (2005). Cross-cultural adaptation and validation of the Korean version of the EQ-5D in patients with rheumatic diseases. Qual Life Res.

[25] Kopec JA, Willison KD (2003). A comparative review of four preference-weighted measures of health-related quality of life. J Clin Epidemiol.

[26] Al Zoubi FM, Preuss RA (2013). Reliability of a measure of total lumbar spine range of motion in individuals with low back pain. J Appl Biomech.

[27] Edwards IR, Aronson JK (2000). Adverse drug reactions: definitions, diagnosis, and management. Lancet.

[28] Spilker B. Quality of life and pharmacoeconomics in clinical trials. Philadelphia: Lippincott Williams & Wilkins. 1995;1312.

